# TCM “medicine and food homology” in the management of post-COVID disorders

**DOI:** 10.3389/fimmu.2023.1234307

**Published:** 2023-08-31

**Authors:** Chester Yan Jie Ng, Hung Hung Bun, Yan Zhao, Linda L. D. Zhong

**Affiliations:** ^1^ School of Biological Sciences, Nanyang Technological University, Singapore, Singapore; ^2^ The University of Hong Kong (HKU) School of Professional and Continuing Education, Hong Kong, Hong Kong SAR, China; ^3^ School of Chinese Medicine, Hong Kong Baptist University, Hong Kong, Hong Kong SAR, China; ^4^ Guangdong-Hong Kong-Macau Joint Lab on Chinese Medicine and Immune Disease Research, Hong Kong, Hong Kong SAR, China

**Keywords:** post-COVID, long-COVID, medicinal food, Chinese medicine, herbs

## Abstract

**Background:**

The World Health Organization declared that COVID-19 is no longer a public health emergency of global concern on May 5, 2023. Post-COVID disorders are, however, becoming more common. Hence, there lies a growing need to develop safe and effective treatment measures to manage post-COVID disorders. Investigating the use of TCM medicinal foods in the long-term therapy of post-COVID illnesses may be beneficial given contemporary research’s emphasis on the development of medicinal foods.

**Scope and approach:**

The use of medicinal foods for the long-term treatment of post-COVID disorders is highlighted in this review. Following a discussion of the history of the TCM “Medicine and Food Homology” theory, the pathophysiological effects of post-COVID disorders will be briefly reviewed. An analysis of TCM medicinal foods and their functions in treating post-COVID disorders will then be provided before offering some insight into potential directions for future research and application.

**Key findings and discussion:**

TCM medicinal foods can manage different aspects of post-COVID disorders. The use of medicinal foods in the long-term management of post-COVID illnesses may be a safe and efficient therapy choice because they are typically milder in nature than chronic drug use. These findings may also be applied in the long-term post-disease treatment of similar respiratory disorders.

## 1 Introduction

In December 2019, an outbreak of pneumonia of unknown origin was reported in Wuhan, Hubei Province, China. Thereafter, a novel respiratory coronavirus was isolated, and it was named as severe acute respiratory syndrome coronavirus 2 (SARS-CoV-2) (1). Due to the high infection rate of the virus, the World Health Organization (WHO) Emergency Committee declared a global health emergency based on growing case notification rates at Chinese and international locations (2). Finally, after three years, WHO announced on 5^th^ May 2023 that COVID-19 is no longer a public health emergency of international concern, following a recommendation by the organization’s COVID-19 emergency committee (3).

However, the prevalence of post-COVID disorders is gradually on the rise (4). Post-COVID disorders are defined as a heterogeneous clinical syndrome involving multiple organ systems and encompassing pulmonary and extrapulmonary disruption (5). It is further characterized by the persistence of symptoms, usually for 3 months or more, and for at least 2 months, following an acute SARS-CoV-2 infection that cannot be explained by an alternative diagnosis (6). Post-COVID disorders have been found to occur in about 10%-30% of individuals infected by SARS-CoV-2 and has recently been proposed to cause neurologic symptoms in 30% of those infected (7). Some other symptoms that might arise could include cardiovascular, respiratory, digestive, and excretory complications (8, 9). Although the exact mechanisms of action have not been confirmed in such short time, some current hypotheses include (i) persistent virus or viral antigens and RNA in tissues that drive chronic inflammation, (ii) the triggering of autoimmunity after acute viral infection, (iii) dysbiosis of microbiome or virome, and (iv) unrepaired tissue damage (10, 11). Hence, there is a need to develop safe and effective treatment alternatives to manage post-COVID disorders in the long run.

“Medicine and Food Homology” (MFH) is a concept introduced by Traditional Chinese Medicine (TCM) (12, 13). This idea has a long history since our forefathers saw the manufacture of TCM medical items and food as one and the same. This is mostly owing to the shared roots of both TCM medical products and foods, and if utilized in conjunction, this could result in several health benefits (13). MFH materials that fall under this category are characterized by their concurrent usage as both food and medicine, thus forming the basis of food therapy (14). In addition to their ability to treat specific disorders, MFH materials are widely employed in disease prevention due to their ease of incorporation into our everyday meals (15). In terms of safety, the side effects of food consumption are relatively mild, while drug or medicine consumption possesses a higher risk of unwanted side effects (16). Hence, it could be useful to explore the integration of MFH into the diets of patients with post-COVID disorders, or as a means of disease prevention upon recovery from COVID-19.

To the best of our knowledge, a thorough assessment of the use of MFH in the treatment of post-COVID illnesses has not yet been published. Reviews that are now available cover topics like the usage of medications to treat post-COVID problems or the application of TCM to treat COVID-19 (8, 17, 18). Therefore, we have decided to review both the mechanisms of action of MFH medicinal foods and post-COVID illnesses to investigate integrating MFH into the treatment of post-COVID disorders.

Hence, this review aims to provide a brief introduction to the concept of “Medicine and Food Homology”, before summarizing the pathophysiological factors and clinical disorders arising post- COVID. We also aim to explore the usage of medicinal foods and their mechanisms of action in the management of post-COVID disorders and its complications.

## 2 “Medicine and food homology”

### 2.1 Concept

According to TCM theory, MFH refers to an overlapping relationship between food and drugs, whereby the use of TCM as a food product or a drug is largely dependent on its dosage (14). TCM herbs with higher toxicity should be consumed in smaller amounts, and the vice versa is true. Hence, MFH herbs are usually low in toxicity and can be consumed in larger amounts as a source of food. In recent years, the Chinese Ministry of Health has also released a list of material which can be “used as both food and drugs”, thus exemplifying the concept of MFH (13). This list is ever-growing as research progresses, and today, there are over 100 types of herbs that are recognized as MFH materials (14).

### 2.2 Relationship between TCM and food

TCM and food are intimately associated since both of their sources, which are found in nature, are tied to each other. Materials taken in vast quantities as part of a person’s diet are referred to as foods, whereas substances with better therapeutic properties are referred to as medications or medicine (19). The class of materials known as MFH includes those that serve both dietary and therapeutic purposes, proving that food and medicine are not mutually exclusive. Walnuts, for instance, are frequently utilized as ingredients in the preparation of foods like desserts or for snacking. Walnuts do, however, also have a medicinal benefit in TCM to enhance bowel movement (20). Hence, this example highlights the close relationship between TCM and food. In addition, TCM medical food therapy advocates maintaining a well-balanced diet according to one’s age, gender and constitution, which is in line with the point of views of nutritionists (21). According to nutritionists, a balanced diet should include well-balanced levels of carbohydrate, protein, and fat to ensure our bodies have enough basic metabolites like glucose and amino acids to support optimal bodily function (22). Similarly, TCM advocates for a well-proportioned diet which should consist of a good balance of grains, fruits, livestock, and vegetables to maintain the normal function of human body (23). Therefore, we can draw parallels between TCM medical food therapy and nutrition, thus showing the potential of TCM medical food therapy in disease management.

**Figure 1:**
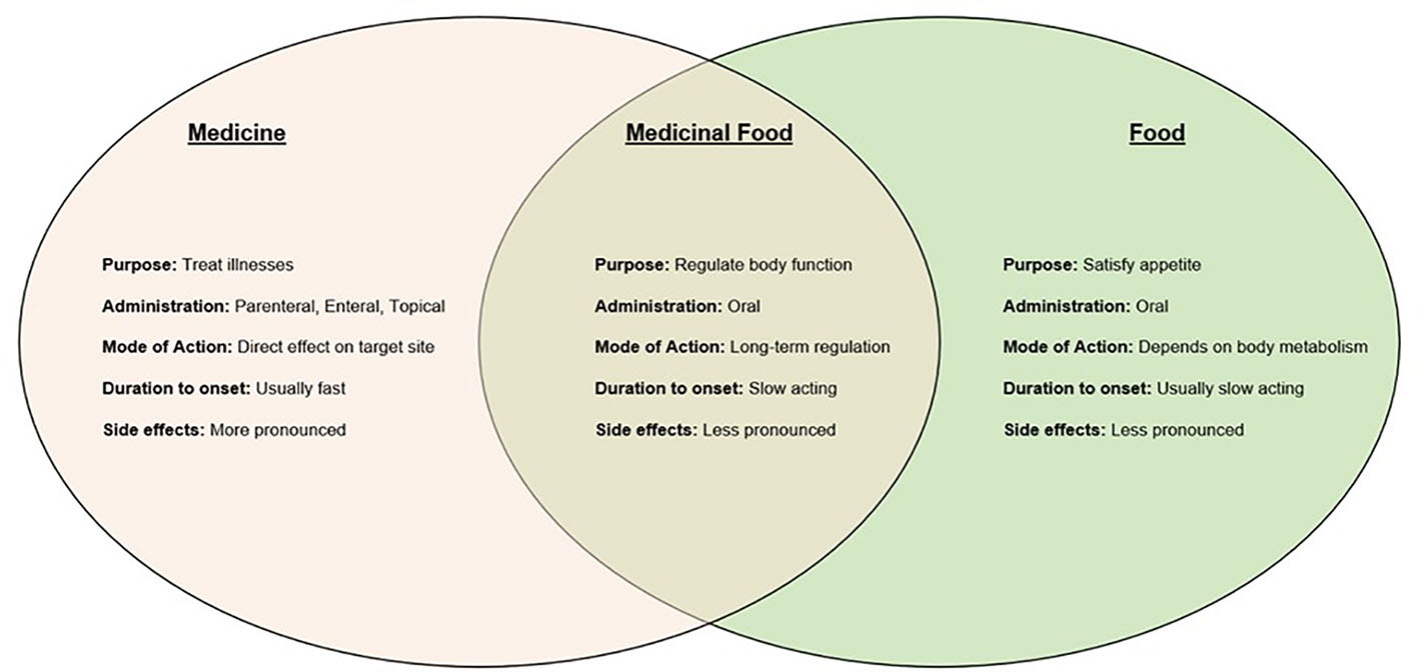
Key relationships between TCM and food.

However, there are differences between TCM and food. Some factors to consider include the purpose of consumption, mode of administration, mode of action, duration to onset of effects, and the visibility of side effects. Therefore, even though they serve different purposes, TCM and food are tightly intertwined. The dosage or amount is the key to the variances. TCM as a medicine has the potential to have strong effects in little doses to treat conditions that need urgent medical attention. On the other hand, they could also have a longer-lasting and slower-acting effect when consumed as a medicinal food, which could be useful in the treatment of chronic disorders, which usually develop gradually (24). **Figure 1** below summarizes key relationships between TCM and food.

As a result, TCM is a strong therapeutic choice due to its adaptability. A balanced diet has also been proven in studies to be crucial for minimizing micronutrient deficiencies and preventing viral infections such as SARS-CoV-2, and in the case of post-COVID issues, which develop gradually, perhaps the longer-term advantages of medicinal foods will prove effective in treating them (25).

## 3 Pathophysiology of post-COVID disorders

SARS-CoV-2 infection affects multiple body systems as the main target for SARS-CoV-2 binding and infection, the angiotensin-converting enzyme 2 (ACE2) receptor, is abundant in cells of most organs (26). The binding of SARS-CoV-2 to the ACE2 receptor on the cell surface reduces ACE2 abundance and increases Angiotensin II levels while decreasing Ang 1-9 and Ang 1-7, which can cause inflammation, oxidative stress, fibrosis, thrombosis, cell proliferation, salt and water retention, and vasoconstriction, thus resulting in cell injury (27, 28). As ACE2 is closely implicated with higher risks of cardiovascular complications and development of cardiovascular diseases, dysfunction of ACE2 after virus binding and dysregulation of the renin-angiotensin-aldosterone system (RAAS) signaling may worsen the outcomes of people affected by COVID-19 and with preexisting cardiovascular diseases (29). Most patients experience a CD4 and CD8 cellular response as well as a regulated inflammatory response, and they recover quickly. Immune dysregulation coupled with high levels of cytokines interleukin-1 (IL-1), IL-6, IL-2, and IL-10 occurs in individuals with more severe infection, hence resulting in the condition “cytokine storm” (30).

Although the exact pathophysiological mechanisms of post-COVID syndrome are not yet clearly elucidated, persistent inflammation is thought to be a crucial pathogenic component. According to studies, post-COVID patients experienced a hyper-inflammatory acute phase with significantly raised C-reactive protein CRP and IL-6 levels (31). An increase in circulating cytokines, such as IL-6, which may cross the blood-brain barrier, could lead to central nervous system (CNS) disorders, which may eventually affect other organ systems as well (26). Furthermore, SARS-CoV-2-mediated ACE2 failure might result in Angiotensin II buildup, reducing the capacity to counteract the RAAS pathway activation and contributing to the development of cardiovascular-related complications (29). In addition, viral persistence of SARS-CoV-2 has been postulated to be a key driver behind post-COVID syndrome. Persistent virus reservoirs have been found in stool samples post SARS-CoV-2 infection (32). It has also been shown that at a year after SARS-CoV-2 infection, the SARS-CoV-2 protein spike was detected in 60% of patients with post-COVID syndrome and not in controls and the greater the number of organ systems involved in symptoms, the greater was the amount of detectable protein spikes (33). Hence, this further implies that post-COVID symptoms could be due to some form of uncleared viral reservoir.

**Figure 2:**
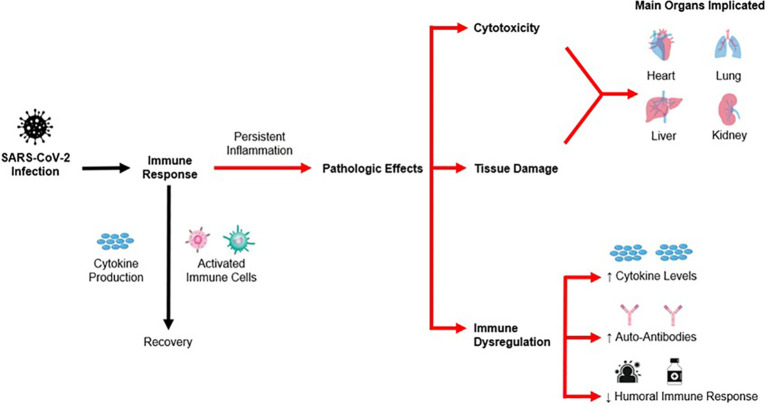
Pathophysiological effects of post-COVID disorders.

The pathophysiological effects of post-COVID disorders can be split into three main stages, namely (1) direct cytotoxic effects, (2) immunological effects and (3) apoptosis and injury to tissue and organs. The sections below will elaborate on each of the following stages, and a summary of the pathophysiology is illustrated in **Figure 2** below.

### 3.1 Direct cytotoxic effects

Pathogen-associated molecular patterns (PAMPs) are produced during the early stages of SARS-CoV-2 infection. PAMPs have immunogenic qualities and can cause a chain reaction of immunological and metabolic alterations in several physiological systems (34). Some examples of immunopathological patterns include an increased content of C-reactive protein (CRP), complement system components C3 and C4 and cytokines (TNF-α, IL-6, IL-10, IL-18) activation, the formation of a wide range of autoantibodies ANA and an overall low efficiency of endocytosis in oxygen-independent phagocytosis (35). As the phagocytic activity reaches its functional limit, activation of neutrophil traps occurs, which can contribute to further induction of molecular cytotoxic patterns, known as damage-associated molecular patterns (DAMPs) (36). As a result, this leads to activation of barrier innate immunity factors and a cytokine storm in some patients in the acute period of SARS-CoV-2, which could eventually result in multiple organ dysfunction syndrome (MODS) (37, 38). Along with DAMPs generation, proteolytic enzymes are triggered, which may result in vascular endothelial inflammation, significant lymphocytic infiltration with complement protein participation, and the creation of a membrane attack complex (39). Therefore, this could result in cytotoxicity, and increased cell damage.

#### 3.1.1 Cytotoxicity in cardiovascular cells

A study by Bojkova et al. has found that SARS-CoV-2 infects and induces cytotoxic effects in human cardiomyocytes (40). In an *in vitro* setting, it was discovered that SARS-CoV-2 infection of cardiomyocytes is dependent on both ACE2 and cathepsin. Viral infection also displayed cytotoxic effects and suppression of cardiomyocyte beating, thus implying that SARS-CoV-2 infection may have a long-term negative impact on the human heart. Similarly, in a case study presented by Gauchotte et al, it was discovered that viral infection of cardiomyocytes could induce direct cell damage, but also indirect damage through a specific T cell cytotoxic immune response (41). The case study being investigated had a high proportion of CD8+ TIA1+ T lymphocytes, with half of these cytotoxic cells activated, characterized by an expression of granzyme B.

A study by Pannucci et al. also proposed a mechanism in which heart-produced hepatocyte growth factor (HGF) could induce T-cell cardio-tropism and directly promote the recruitment of these activated T lymphocytes to the heart, where they could elicit cell-mediated cytotoxicity and destruction of myocardial tissue by interacting with the receptor tyrosine kinase c-MET expressed on naive T lymphocytes (42). T-lymphocyte-mediated cytotoxicity and subsequent lysis of infected cardiomyocytes may arise from recognition of the SARS-CoV-2 antigen presented by major histocompatibility complex class I (MHCI) on the surface of infected cardiomyocytes (43).

Collectively, these studies suggest that SARS-CoV-2-induced fulminant myocarditis could be induced by a direct infection of cardiomyocytes associated with an intense cytotoxic T cell immune response.

#### 3.1.2 Cytotoxicity in pulmonary cells

In lung tissue, histologic assessment revealed that diffuse alveolar damage (DAD) and micro-thrombosis were the most common histologic finding (44). Pneumocyte necrosis, desquamation and hyperplasia with fibrin deposition, and interstitial inflammation were the effects observed. Immunohistochemical findings also suggested perivascular aggregation and diffuse infiltration of alveolar walls by CD4+ and CD8+ T lymphocytes.

At a cellular level, a study by Cheon et al. has shown that tissue resident cells from the respiratory tract expressed higher levels of NKG7, a cytotoxic molecule that can promote lethal inflammation after infection and granzyme K, an inflammatory granzyme that promotes fibroblast activation (45–47). Clonally expanded T cells from the bronchoalveolar lavage fluid (BAL) were also shown to contain higher quantities of effector or cytotoxic molecules, indicating that they were likely antigen-experienced T cells and correlated with lower lung function and worse lung pathology. An increase in cytotoxic T cells in the BAL is associated with an increased risk of epithelial damage and airway diseases (48). Another study by Kaneko et al. has also demonstrated that cytotoxic CD4+ T cells increase significantly in the lungs, draining lymph nodes and blood as COVID-19 progresses (49).

Collectively, these studies demonstrate SARS-CoV-2-induced cytotoxicity in pulmonary cells has negative impacts on lung functions in the long run.

#### 3.1.3 Cytotoxicity in hepatocellular cells

SARS-CoV-2 liver damage is caused by direct cytotoxicity of SARS-CoV-2 virus replication in the liver, which is caused by factors such as severe systemic inflammatory response syndrome (SIRS) in COVID-19, hypoxic conditions, vascular changes, drug-induced liver injury (DILI), and previous exacerbations of liver disease (50). Analysis also showed elevated levels of proinflammatory CCR6+Th17 in CD4 T cells and cytotoxic granulations in CD8 cells (51).

A previous study by Banales et al. has also shown that in COVID-19 patients, there was elevated ACE2 expression in cholangiocytes and hepatocytes (52). Cholangiocyte structural and functional abnormalities could result in impaired bile production, inflammation, fibrosis, and liver dysfunction, resulting in an overall increase in ACE2 expression in liver tissue, which could be one of the mechanisms of liver damage induced by SARS-CoV-2 infection.

Collectively, patients infected with SARS-CoV-2 may develop hypoxic-ischemic liver injury, thus affecting the regulation of hepatocellular ACE2 expression (50, 53). As a result, extrapulmonary SARS-CoV-2 occurs and complications such as heart failure and hepatic congestion may arise (54).

#### 3.1.4 Cytotoxicity in renal cells

The SARS-CoV-2 virus has been shown to affect the renal cells via direct cytotoxicity (55). Due to abundant expression of ACE2 in the kidney, which is used by SARS-CoV-2 to enter host tissue, this could be a possible reason to account for increased cytotoxic effects in renal cells (56).

The kidney’s tubulo-interstitial compartment was also discovered to be a major location of cytotoxicity. A possible mechanism of tubular injury is direct cytotoxic action of the virus in the tubules, resulting in mitochondrial malfunction, acute tubular necrosis, tubular proteinuria, and hematuria (56).

### 3.2 Immunological dysregulation

Overall, one common trend amongst post-COVID patients was elevated cytokine levels. Inflammatory cytokines such as IL-6, TNF-α, and IL-1β were found to be elevated in post-COVID patients (57). IFN- β and IFN-λ1 levels also remained elevated 8 months after infection in post-COVID patients compared with recovered controls (58). Hence, the persistence of viral components could result in chronically elevated IFNs and cytokine levels, thus leading to common post-COVID symptoms such as persistent low-grade fever.

Another trend observed was the high levels of auto-antibodies (autoAbs) in post-COVID patients (59). A study found that G-protein coupled receptor (GPCR) functional autoAbs were found in post-COVID patients. These autoAbs could either activate their target receptors, causing a positive chronotropic effect in neonatal rat cardiomyocytes or cause a negative chronotropic effect (60). Such autoAbs stimulation could affect the cardiovascular, pulmonary, and the central nervous systems, which could have contributed to the pathogenesis of post-COVID disorders (61). Furthermore, an examination of antinuclear autoAbs utilizing 102 clinically mapped auto-antigens revealed a link between antinuclear antibodies such U1-snRNP and anti-SS-B/La with residual symptoms and inflammation 12 months after acute SARS-CoV-2 infection (59).TNF-α, D-dimer and IL-1β were also primarily responsible for persistent inflammation in post-COVID patients. Hence, autoAbs can be induced via SARS-CoV-2 infection, which could subsequently play a role in the persistence of post-COVID syndrome as well.

Lastly, dysregulated immune response was a recurring characteristic of post-COVID patients (62). In post-COVID patients, decreased concentration of IgG3 subclass immunoglobulins was observed, which constitute the predominant antibody responses against COVID -19 (63). The presence of anti-idiotype antibodies against SARS-CoV-2 SIgG has also recently been proposed as a mechanism of downregulation of the specific humoral response by binding to protective neutralizing antibodies, resulting in immune complex formation and clearance (64). Hence, an impaired humoral immune response could then result in persistent COVID -19 infection or the formation of an antigenic reservoir, giving rise to immune stimulation, sustained inflammation and autoreactivity (65, 66).

### 3.3 Apoptosis and injury to tissue and organs

SARS-CoV-2 can directly infect vascular endothelial cells (ECs), with subsequent systemic endotheliitis and cellular apoptosis (67, 68). ECs are important cells in our body, and are responsible for the regulation of key physiologic processes such as blood rheology, vasomotor tone regulation, osmotic balance, and vascular barrier function (69, 70). The degree of apoptosis of pulmonary microvascular endothelial cells in patients infected with SARS-CoV-2 has also been shown to correlate with disease severity (71). With a loss of function of ECs, this could give rise to a host of vascular complications, which could pose serious health concerns in the future. Endothelial cell damage followed by activation are considered key steps in thrombus formation and studies have shown that early post-mortem acute infection showed microthrombi of the small myocardial vasculature in most cases (72). Therefore, COVID-19-associated coagulopathy is a critical component of pathogenesis, especially on endothelial cells (73). The persistence of aberrant levels of sustained endotheliopathy and hypercoagulability after acute SARS-CoV-2 infection may thus correlate with the induction of persistent post-COVID symptoms (74).

It was also revealed that patients with post-COVID disorders had considerably greater levels of expression of the markers CD95 and CD54 (75). Apoptosis is marked by CD95, while intracellular adherence is marked by CD54 (76, 77). As a result, these findings point to a persistently high level of dysfunctional immune response in the short term after recovery, and the expression of the intercellular adhesion molecule demonstrates the involvement of the vascular wall endothelium in the inflammatory process as one of the mechanisms of post-COVID syndrome pathogenesis.

#### 3.3.1 Injury to cardiovascular system

According to histological assessment, it was postulated that perivascular infiltration of T lymphocytes might have played a role in myocardial injury by disrupting microvascular perfusion and/or T lymphocyte cytotoxic effects (44).

Microthrombi are another typical histopathologic finding in post-COVID patients’ heart tissue (78). Although micro thrombosis is a normal physiological occurrence, if it is disrupted by immune responses, as in the case of severe SARS-CoV-2 infection, it can lead to significant cardiac necrosis. Pathologic features such as increased systemic inflammatory biomarkers, significant neutrophil infiltration, and clot composition are also detected in this case.

#### 3.3.2 Injury to respiratory system

Diffuse alveolar damage (DAD) was observed, including exudative, proliferative, and fibrotic, suggesting ongoing cycles of lung damage, likely arising from persistent viral replication in the lung tissue and subsequent T cell over-activation and cytokine storms (44). The presence of micro-thrombi was also observed in the pulmonary microvasculature, which might have resulted from a systemic hypercoagulable state.

Pulmonary fibrosis is also a common occurrence in patients post-COVID, especially after acute respiratory distress syndrome (ARDS) (79). It is characterized by chronic inflammation, leading to lowered protective lung ventilation function. Pathological characteristics include DAD which is characterized by an initial (acute inflammatory) exudative phase with edema, hyaline membranes, and interstitial acute inflammation, followed by an organizing phase with loose organizing fibrosis mostly within the alveolar septa, and type II pneumocyte hyperplasia (80).

#### 3.3.3 Injury to digestive system

Histological assessment revealed central perivenular necrosis and neutrophilic lobular micro-abscess formation, along with sinusoidal lymphocytosis (44). The related micro-vesicular steatosis, arising from acquired cellular oxidative defects, can also be a detrimental prognostic feature (81). The pathologic findings were also noted to be like that observed in patients with influenza H1N1, Cytomegalovirus, Epstein-Barr Virus, or drug-induced hepatitis.

#### 3.3.4 Injury to urinary system

Histological assessment of kidney tissue revealed acute tubular injury, such as necrosis and flattening of tubular epithelium, multiple tubular casts, and interstitial oedema, on top of chronic changes including interstitial nephritis and tubular atrophy (44). These findings correlate with past findings that persons with chronic renal disease are more susceptible to severe infection by SARS-CoV-2 (82).

## 4 Role of medicinal foods in managing post-COVID complications

**Figure 3:**
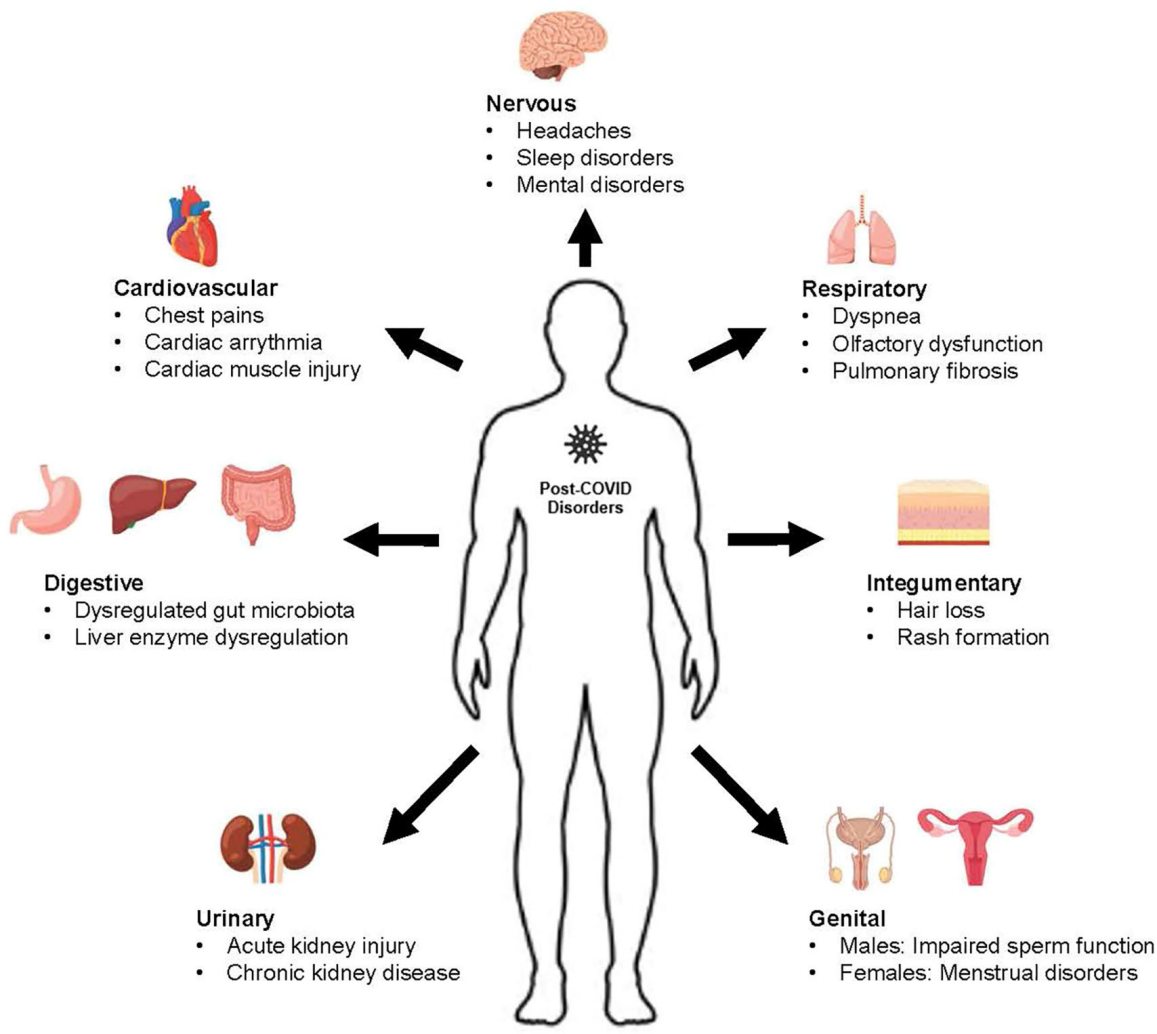
Summary of Post-COVID disorders by body system.

Post-COVID complications have been found to implicate many body systems, which include the cardiovascular, respiratory, urinary, nervous digestive, genital, and integumentary systems. The complications regarding the individual body systems are elaborated in the sections below, and a summary diagram of the is shown in **Figure 3** below. A list of potential TCM medicinal foods and their mechanism of action is also summarized in **Table 1** below.

**Table 1:** Summary of potential TCM medicinal foods to manage post-COVID disorders .

Name	Main Active Ingredients	Consumed Part	Bioactivities	Body system(s) targeted	Potential clinical benefits	References
***Angelica dahurica***	Coumarins, volatile oils, polysaccharides	Root	Analgesic, anti-inflammatory, anti-tumor, anti-depressant, and antioxidative	Integumentary	↓ Skin itching ↓ Pain and swelling	(83, 84)
***Armeniaca vulgaris***	Amygdalin	Seed	Antioxidative, anti-tumor	Integumentary	↓ Acne formation ↑ Skin moisture	(85–87)
***Astragalus membranaceus***	Polysaccharides	Root	Immunomodulation, antioxidative, antitumor, anti-diabetes, antiviral, hepatoprotection, anti-inflammatory, anti-atherosclerosis, and neuroprotective	Nervous Respiratory Cardiovascular Urinary	↑ Neurological protection ↑ Respiratory health ↑ Cardiovascular protection ↑ Renal protection	(88–90)
***Carica papaya***	Phenolics, flavonoids, alkaloids	Fruit	Anti-inflammatory, hypoglycemic, anti-fertility, abortifacient, hepatoprotective, wound healing, and anti-tumor	Digestive	↑ Gastrointestinal health ↑ Digestion	(91, 92)
***Cassia fistula***	Anthraquinones, anthraquinone glycosides	Seed	Antioxidative, antimicrobial, anti-inflammatory, antidiabetic, anti-tumor, hepatoprotective	Urinary Cardiovascular Respiratory Digestive	↑ Vision ↑ Cardiovascular protection ↑ Respiratory function ↑ Bowel movement	(93–95)
***Cinnamomum verum***	Cinnamaldehyde, camphor	Bark	Antioxidative, antimicrobial, and antidiabetic	Respiratory Nervous Urinary Digestive	↑ Respiratory function ↑ Renal protection ↑ Gastrointestinal health ↑ Neurological function	(96–98)
***Chrysanthemum indicum***	Flavonoids, terpenoids, polysaccharides and unsaturated fatty acids	Flower	Antioxidative, antimicrobial, anti-inflammatory, anti-tumor, anti-allergic, anti-obesity, immune regulation, hepatoprotective and nephroprotective	Cardiovascular Nervous Urinary Digestive	↑ Cardiovascular protection ↑ Renal protection ↑ Gastrointestinal health ↑ Neurological protection	(99, 100)
***Crataegus pinnatifida***	Flavonoids, phenols, terpenoids and polysaccharides	Fruit, flower, leaf	Antioxidative, anti-inflammatory, anti-diabetic, anti-tumor	Cardiovascular Digestive	↑ Cardiovascular protection ↑ Digestion ↑ Appetite	(101, 102)
***Curcuma longa***	Curcumin	Rhizome	Anti-inflammatory, antioxidative, antimutagenic, antidiabetic, antibacterial, hepatoprotective, expectorant and anti-tumor	Cardiovascular Nervous Urinary Digestive Respiratory	↑ Cardiovascular protection ↑ Renal protection ↑ Respiratory health ↑ Gastrointestinal health ↑ Neurological protection	(103)
***Foeniculum vulgare***	Trans-anethole, anethole, fenchone	Fruit	Antibacterial, antifungal, antiviral, antioxidative, anti-inflammatory, antimutagenic, antinociceptive, hepatoprotective, bronchodilatory, and memory enhancing	Digestive	↑ Gastrointestinal health ↑ Bowel movement	(104)
***Folium mori***	Flavonoids, phenolic acids, alkaloids	Leaves	Antioxidative, hypoglycemic, anti-cholesterol (affecting lipid metabolism), anti-obesity, anti-inflammatory, anti-tumor	Cardiovascular Nervous Urinary Respiratory	↑ Cardiovascular protection ↑ Neurological function ↑ Renal protection ↑ Respiratory health	(105, 106)
***Fructus barbarum***	Ascorbic acid, thiamine, and riboflavin	Fruit	Antidiabetic, antiproliferative, preserving retinal function, and antioxidative activity	Nervous	↑ Neurological function ↑ Sleep quality ↓ Fatigue	(107, 108)
***Fructus gardeniae***	Geniposides	Fruit	Hepatoprotective, choleretic, anti-inflammatory, antioxidative, neuroprotective, anti-diabetic, anti-apoptotic and anti-tumor	Nervous	↓ Headaches ↑ Neurological function	(109, 110)
***Gastrodia elata***	Polysaccharides	Rhizome	Anti-tumor, antiviral, antiosteoporosis, antioxidative immunomodulatory, and neuroprotective	Nervous	↓ Blood pressure ↑ Neurological function	(111–113)
***Glycyrrhiza glabra***	Flavonoids, glycyrrhizin	Root, stem	Antibacterial, antioxidative, antimalarial, antispasmodic, anti-inflammatory, anti-hyper glycemic, antiulcer, antiviral, antihepatotoxic, antifungal	Digestive Respiratory Cardiovascular	↑ Gastrointestinal health ↓ Abdominal pain ↑ Respiratory health ↑ Cardiovascular protection	(114, 115)
***Houttuynia cordata***	Essential oils, alkaloids	Leaves	Antioxidative, anti-inflammatory, and antiviral	Respiratory Urinary	↑ Respiratory health ↑ Renal function ↑ Renal protection	(116, 117)
***Hovenia dulcis***	Flavonoids	Fruit	Hepatoprotective, antioxidative, antimicrobial and antidiabetic	Cardiovascular Nervous	↑ Alcohol detoxification ↓ Blood pressure ↑ Neurological protection	(118, 119)
***Illicium verum***	Flavonoids	Fruit	Antioxidative, antimicrobial, antifungal, anthelmintic, insecticidal, secretolytic, antinociceptive, anti-inflammatory, gastroprotective, sedative, expectorant and spasmolytic, and estrogenic	Digestive	↑ Gastrointestinal health ↓ Abdominal pain	(120, 121)
***Lonicera japonica***	Triterpenoid saponins and sapogenin	Flower	Hepatoprotective, anti-inflammatory, anti-bacterial, anti-allergic, immunomodulatory, anti-tumor, molluscicidal	Respiratory	↑ Throat relief	(88, 122, 123)
***Mentha piperita***	Menthol, rosmarinic acid, flavonoids	Leaves	Antimicrobial, antiviral, antioxidative, anti-tumor, antiallergenic	Respiratory	↑ Throat relief	(124)
***Olea europaea***	Polyphenols, glycosides	Leaves, fruit	Antioxidative, hypoglycemic, antihypertensive, antimicrobial, and antiatherosclerotic	Nervous Urinary Digestive	↑ Renal protection ↑ Neurological protection ↑ Gastrointestinal health	(125–127)
***Piper longum***	Alkaloids	Fruit	Anti-amoebic, anthelminthic, anti-tumor and anti-diabetic	Cardiovascular Nervous Urinary Digestive Respiratory	↑ Cardiovascular protection ↑ Renal protection ↓ Abdominal pain ↑ Neurological protection ↑ Renal function	(128, 129)
***Piper nigrum***	Alkaloids	Seed	Antihypertensive, antiplatelet, antioxidative, antitumor, anti-asthmatics, analgesic, anti-inflammatory, anti-diarrheal, antispasmodic, antidepressants, immunomodulatory, anticonvulsant, anti-thyroids, antibacterial, antifungal, hepatoprotective	Cardiovascular Nervous Urinary Digestive	↑ Cardiovascular protection ↑ Neurological function ↑ Renal protection ↑ Gastrointestinal health	(130, 131)
***Phyllanthus emblica***	Phenolic acids	Fruit	Antidiabetic, hypolipidemic, antibacterial, antioxidant, antiulcerogenic, hepatoprotective, gastroprotective	Cardiovascular Nervous Urinary Digestive Respiratory	↑ Cardiovascular protection ↑ Neurological protection ↑ Renal function ↑ Throat relief ↑ Appetite	(132, 133)
***Poria cocos***	Polysaccharides, triterpenoids	Sclerotium	Anti-tumor, anti-inflammatory, antioxidative, and antiviral	Integumentary Nervous Digestive	↓ Acne formation ↑ Neurological function ↑ Gastrointestinal health	(134, 135)
***Rubus chingii***	Phenolic acids, terpenoids, flavonoids	Fruit	Antioxidative, anti-inflammatory, and anti-tumor	Genital	↑ Reproductive health	(136, 137)
***Portulaca oleracea***	Flavonoids, alkaloids, fatty acids	Whole plant	Neuroprotective, antimicrobial, antidiabetic, antioxidative, anti-inflammatory, antiulcerogenic, and anticancer	Integumentary	↓ Acne formation ↑ Skin moisture	(138, 139)
***Prunus mume***	Phenolics, flavonoids, organic acids	Fruit	Antidiabetic, hepatoprotective anti-tumor, anti-inflammatory and antimicrobial	Integumentary Respiratory	↑ Skin moisture ↑ Throat relief	(140)
***Rhizoma dioscorea***	Polysaccharides, dioscin, diosgenin, allantoin, alkaloids, phenolics	Rhizome	Antioxidative, anti-inflammatory, gastrointestinal protection, gut microbiota regulation, hypoglycemic activity, anti-tumour, and oestrogen-like effect	Urinary Digestive	↑ Gastrointestinal health ↑ Renal protection	(141, 142)
***Siraitia grosvenorii***	Triterpene glycosides	Fruit	Antidiabetic, anticarcinogenic, antibacterial, antioxidative, and antiallergic	Respiratory	↓ Cough ↑ Throat relief	(143–145)
***Sterculiae lychnophorae***	Polysaccharides	Seed	Anti-inflammatory	Respiratory Digestive	↑ Throat relief ↑ Bowel movement	(146, 147)
***Syzygium aromaticum***	Essential oils	Flower bud	Antiseptic, analgesic, antiplatelet, antithrombotic, chemoprotective, antipyretic	Nervous Urinary Digestive	↑ Gastrointestinal health ↑ Neurological function ↑ Renal protection	(148–150)
***Torreya nucifera***	Seed oils	Seed	Antioxidative, anti-inflammatory, anti-nociceptive	Digestive	↑ Bowel movement ↑ Gastrointestinal health	(151–153)
***Zingiber officinale***	Sesquiterpenoids, zingiberene	Rhizome	Antioxidative, anticancer, anti-inflammatory, anti-apoptotic, anti-hyperglycemic, anti-hyperlipidemic and anti-emetic	Cardiovascular Nervous Urinary Digestive	↑ Gastrointestinal health ↓ Abdominal pain ↑ Cardiovascular protection ↑ Renal protection ↑ Neurological protection	(154, 155)

↑, Decreased; ↓, Enhanced.

### 4.1 Cardiovascular system

Cardiovascular complications are a common occurrence in patients infected with SARS-CoV-2. According to a study by the University of Frankfurt, it was discovered that amongst COVID-19 patients, 78% of patients exhibited cardiac issue and 60% of patients exhibited cardiac inflammation, with severe cases showing elevated troponin levels (156). As a result, this leads to a series of complications including arrythmia, cardiac muscle diseases and heart failure (157, 158). Furthermore, other symptoms such as continuing palpitations and chest pain were observed up till 6 months after recovery (38, 159).

Therefore, one of the primary treatment goals is to reduce inflammation. *Curcuma longa* is one such therapeutic food with anti-inflammatory qualities (103). Curcumin, extracted from *Curcuma longa*, was discovered to inhibit cytokine production, specifically IL-1, IL-6, pro-inflammatory cytokines, and TNF-α, and therefore to have anti-inflammatory effect in COVID-19 patients (160). Similarly, glycyrrhizic acid and glycerrhitinic acid produced from *Glycyrrhiza glabra* have been demonstrated to have anti-inflammatory properties similar to glucocorticoids and mineralocorticoids, making them promising candidates for the treatment of inflammatory disorders (114). Flavonoids and triterpenoids from *Glycyrrhiza glabra* also possess cardioprotective abilities (115). *Piper nigrum* extracts were also discovered to decrease TNF-α induced NF-κB activation as well as inhibit COX-1 and COX-2 production, demonstrating anti-inflammatory and cardioprotective properties (130). Hence, such medicinal herbs could be helpful in the reduction of inflammation to minimize post-COVID cardiac muscle and cell damage.

Another key treatment goal is the inhibition of the ACE2 receptor and TMPRSS2 protease, which are responsible for mediating SARS-CoV-2 viral entry into the cells (161). In addition to its cardioprotective properties, I-Asarinin, a component of *Piper longum*, was recently discovered to have significantly higher binding activities with ACE2 and TMPRSS2, inhibiting viral entry, which could be useful for the prevention of subsequent re-infections (128).

### 4.2 Respiratory system

As the lungs are the main target of the SARS-CoV-2 virus, pulmonary symptoms are commonly observed in COVID-19 patients. Due to the dysregulated immune response caused by SARS-CoV-2 infection, this increases the production of proinflammatory factors (such as IL-1, IL-9, and IL-8) and inflammatory mediators (such as chemokines and cytokines), which damages the alveolar epithelial cells (46). Hence, alveolar injury results in a reduction in the lungs’ oxygen exchange capacity, resulting in hypoxemia (162). With the vicious cycle of inflammation and alveolar destruction, this could also possibly lead to the onset of ARDS and pulmonary fibrosis (79). Alveolar injuries also cause blood clots to form in COVID-19 patients, leading to problems such as pulmonary embolism and stroke (163). Hence, two main pathologic effects of such viral lung infection are inflammation and pulmonary fibrosis (164). These severe complications could have negative impacts on one’s respiratory health in the long run.

Among the many pulmonary symptoms observed, one of the most common symptoms is dyspnea. Studies have found that dyspnea has been reported in 42% to 66% of cases at 60 to 100 days of follow-up (165). CT scans of patients reporting dyspnea also showed signs of lymphadenopathy, pleural effusion and cavitations (166). Olfactory dysfunction is also another common respiratory symptom observed in post-COVID patients. This symptom is believed to be caused by damage mediated by viral invasion of ACE2 and TMPRSS2 receptors on cells in the nasal and olfactory epithelium (167, 168). In most cases, olfactory dysfunction resolves in about two weeks, with a mean recovery duration of 9 days (169). However, some patients develop olfactory dysfunction which could persist for an extended period (170).

One of the main treatment goals of respiratory disorders is the management of inflammatory cytokine levels. *Astralagus membranaceus* and *Lonicera japonica* extracts have been demonstrated to upregulate selected microRNAs, lower SARS-CoV-2 pathogenesis, and inhibit pro-inflammatory cytokines IL-6 or TNF-α, all of which are important pathogenic contributors in cytokine storms (88). Additionally, *Lonicera japonica* has been shown to inhibit ACE2 binding and viral entry, which could alleviate the symptoms of olfactory dysfunction as well (122). Mogroside IIIE, a triterpenoid glycoside found in *Siraitia grosvenorii*, was also found to inhibit lung fibroblast collagen production by blocking inflammatory-induced direct differentiation of lung pericytes and resident fibroblasts, making it an effective pulmonary fibrosis inhibitor (143, 144). *Sterculiae lychnophorae* has also been used for a long time to treat inflammatory respiratory disorders such as pharyngitis by inhibiting histamine, serotonin, bradykinin, and prostaglandins, all of which are major mediators of acute inflammation (146). *Folium mori* also contains rutin, choline, and folic acid, all of which possess anti-inflammatory properties that can help with respiratory tract inflammatory conditions (105).

Another main treatment goal is to reduce the presence of reactive oxygen species (ROS), which causes lung injury. Extracts of *Mentha piperita* and *Houttuynia cordata* have been reported to be high in antioxidants, which scavenge free radicals and minimize lung cell damage (116, 124). Furthermore, *Houttuynia cordata* also possesses anti-inflammatory action and modulates the release of inflammatory factors to reduce lung damage (117).

### 4.3 Urinary system

Renal conditions are also commonly reported in patients recovering from SARS-CoV-2 infection. During the infection, the SARS-CoV-2 virus infiltrates the kidney and binds to ACE2, thus damaging renal resident cells, and causing angiotensin dysregulation, innate and adaptive immune pathway activation, and hyper-coagulation (171). Some resulting complications include acute kidney injury (AKI), electrolyte disturbances, and renal replacement therapy (RRT) (172). Amongst these complications, AKI is a prominent cause of COVID-19-related death (173). AKI could also lead to long-term renal complications in patients such as microalbuminuria and chronic kidney diseases, thus requiring intensive care such as routine dialysis (174).

Hence, one of the primary treatment goals is to minimize renal inflammation. *Olea europaea* is one medicinal plant that can accomplish this. Oleuropein, a glycosylated secoiridoid found in *Olea europaea* fruit and leaves, is a kind of phenolic bitter molecule that has the capacity to suppress inflammatory cytokines (IL-6, TNF-α, and IL-1) and NF- κB activation. As a result, this increases anti-inflammatory actions (125). Similarly, *Rhizoma dioscorea* has been shown to reduce IL-1β, IL-6, and TNF-α levels to reduce renal inflammation (141).

Additionally, inhibiting ACE2 is also critical in the treatment of urinary related post-COVID diseases. *Syzygium aromaticum* and *Cinnamomum verum* were discovered to inhibit SARS-CoV-2 spike protein binding to ACE2 and reduce ACE2 function in a dose-dependent manner (96, 148).

### 4.4 Nervous system

Neurological problems have also been described in SARS-CoV-2-infected individuals. Infection with SARS-CoV-2 has been demonstrated to reduce the amount of ACE2 receptors in the brain stem, resulting in the death of neurons and the loss of function of many baroreceptors (175). Additionally, elevated cytokine levels post-COVID were also correlated to the occurrences of migraine-like and late-onset headaches (176, 177). After COVID-19 recovery, complications such as auditory and visual hallucinations, schizophrenia, PTSD, and epilepsy were also documented (17). Studies have also found a significant frequency of sleep and mental disorders in patients following COVID-19 recovery (38, 178). In addition, patients infected by SARS-CoV-2 are challenged by severe stressors, including fear of death from life-threatening illness, pain from medical interventions, endotracheal intubation, limited ability to communicate, and the feeling of losing control (179). In some instances, these neurological manifestations can be calamitous and can even lead to death.

“Parkinsonism” is also a common occurrence reported in individuals who have recovered from SARA-CoV-2 infection. SARS-CoV-2 has been detected in the brain and the cerebrospinal fluid of affected patients (180). The dynamic pro-inflammatory state of COVID-19 accompanies abnormal accumulation of α-synuclein in nerve fibers, neurons, and glial cells, which leads to increased oxidative stress and causes neuro-inflammation and Parkinson’s disease symptoms (181, 182). Hence, this leads to patients displaying behaviors such as including progressive rigidity, bradykinesia, postural instability, oculomotor abnormalities and cognitive impairment, despite not being diagnosed with Parkinson’s disease (183).

Vertigo is another symptom reported in post-COVID patients. It is believed to be attributed to the neuroinvasive potential of the SARS-CoV-2 virus (184). Although the exact mechanisms of action are not yet well elucidated, the onset of post-COVID vertigo is hypothesized to be complex, with elements such as excessive inflammatory response, neurotransmission abnormalities, chronic virus damage, and functional modifications all playing a role (185, 186). Furthermore, because the SARS-CoV-2 virus has the potential to target the inner ear, which is particularly vulnerable to ischemia, this might result in vascular problems, induced hypercoagulability, and the production of microthrombi in the inner ear, leading to severe ear injury (187). Hence, the development of vertigo should be addressed in a safe and effective manner to avoid long-term harm to the patients.

Hence, one important therapy objective is to minimize neurological inflammation. *Hovenia dulcis* has been proven to have neurological anti-inflammatory properties in the brain by inhibiting p38MAPK, iNOS, and Nrf2 (118). Similarly, extracts of *Phyllanthus emblica* have been demonstrated to inhibit acetylcholinesterase and butyrylcholinesterase, thus showing anti-inflammatory and neuroprotective properties (132). In *in vivo* models, *Chrysanthemum indicum* extracts have also shown anti-inflammatory properties, reducing neuronal damage and ischemia complications (99).

To manage Parkinsonism, one well-studied medicinal food is *Fructus barbarum*. *Fructus barbarum* has long been known for its antioxidant capabilities, and its antioxidative characteristics have been demonstrated to promote neuroprotection and neuroplasticity, both of which are important in the treatment of neurological illnesses such as Parkinsonism (107). Another important therapeutic objective in the treatment of Parkinsonism is to reduce the excessive buildup of α-synuclein. In animal models, geniposide from *Fructus gardenia* was reported to diminish α-synuclein via disrupting protein interactions (109). Furthermore, geniposides were discovered to exhibit antidepressant properties, which may be effective in treating depressive symptoms in post-COVID patients (110).

Lastly, *Gastrodia elata* could be an effective medicinal food to manage post-COVID vertigo. Gastrodin, a bioactive molecule derived from *Gastrodia elata*, has been used as an injectable for vertigo therapy and has been proved to be safe and effective in many clinical trials (188). In addition, *in vivo* studies have shown that *Gastrodia elata* extract exhibited anti-depressive effects, improved cognitive impairment, and regulated gut microbiome and metabolite regulation (111). Furthermore, *Gastrodia elata* extracts have been demonstrated to alleviate Parkinson Disease symptoms by protecting dopaminergic neurons, lowering α-synuclein buildup, and decreasing neuroinflammation, which might be useful in Parkinsonism management (189). Collectively, *Gastrodia elata* has the potential to be used as a medicinal food to treat post-COVID neurological issues.

### 4.5 Digestive system

Patients who have recovered from SARS-CoV-2 infection have reported experiencing gastrointestinal issues. The presence of the ACE2 receptor in the GI tract may also be a contributing factor to the occurrence of gastrointestinal illnesses (190). Infection by SARS-CoV-2 has been shown to change the gut microbiota, reducing beneficial commensals and hence favoring opportunistic infectious agents (191). For instance, *Faecalibacterium prausnitzii*, which is a beneficial butyrate-producing anaerobe associated with good gut health, was shown to be inversely linked to disease severity in COVID-19 (38). Dysbiosis, visceral hypersensitivity, and increased intestinal permeability are also examples of reported manifestations. These conditions result in insufficient bile acid absorption as well as issues with various metabolic pathways (17). Hence, this could result in the liver being implicated as well, as evidenced by increased levels of enzymes such as higher levels of alkaline phosphatase (ALP), serum glutamic–pyruvic transaminase (SGPT), serum glutamic–oxaloacetic transaminase (SGOT) and bilirubin (192). Some patients have also reported higher serum pro-inflammatory cytokine and chemokine levels (193). In addition, evidence also suggests the possible onset of short-term functional gastrointestinal disorders or dysregulated gut–brain interactions after SARS-CoV-2 infection (194).

*Carica papaya* is one probable therapeutic food that might be useful in the treatment of gastrointestinal issues. *Carica papaya* extracts have been demonstrated to lower gastric juice volume and acidity in a dose-dependent manner, thus exhibiting gastroprotective properties and preventing ulcer development (91). Studies have also shown that a mixture of *Illicium verum* and *Matricaria chamomilla* exerts an anti-motility effect and decreases induced diarrhea in mice, thus showing potential to treat and alleviate gastrointestinal problems (120). Another herb, *Zingiber officinale*, has also been long used in managing chronic gastrointestinal conditions via its antioxidant and anti-inflammatory properties (154). Lastly, *Crataegus pinnatifida* is another popular plant that improves gastrointestinal carbohydrate and fat digestion and absorption, hence improving gastrointestinal function and health (101).

Another key mode of mediating gastrointestinal problems is via the modulation of gut microbiota. *Torreya nucifera* seed oil has been shown to significantly increase the abundance of beneficial bacteria and short-chain fatty acid producers such as *Lactobacillus, Bifidobacterium, Faecalibaculum* and *Allobaculum* (151). *Poria cocos* polysaccharides have also been shown to modulate gut microbiota such as *Akkermansia* and *Faecalibacterium*, which could be beneficial in the long-term management of chronic gastrointestinal disorders (134).

In the event that the liver has been implicated post-COVID, *Cassia fistula* extracts have been shown to decrease ALP, SGOT and SGPT levels, thus improving oxidative stress and hepatic toxicity markers (93). Similarly, *Foeniculum vulgare* and its active component trans-anethole have been shown to boost high-density lipoprotein cholesterol catalase activity and thiol content, as well as improve hepatic toxicity indicators, hence lowering liver damage and fibrosis (104). Furthermore, *Torreya nucifera* seed oil was demonstrated to lower liver and fat indices, total cholesterol, triglycerides, and low-density lipoprotein cholesterol, hence alleviating pathological liver damage induced by a high-fat diet (151).

### 4.6 Genital system

The infection of SARS-CoV-2 could produce post-COVID reproductive complications primarily in males. Infection of Sertoli cells via their ACE2 receptors destroys the seminiferous epithelium barrier of the cells, thus leading to dysfunction of the spermatogenesis cycle (195). This could lead to decreased spermatogenesis and reduced sperm production. In addition, studies have shown that SARS-CoV-2 infection could also result in decreased sperm motility. A study by Valdiva et al. has shown that a decrease of the ACE2 receptor due to the viral attack decreases sperm motility because the ACE2-angiotensin-(1–7)-Mas receptor maintains sperm mobility by activating the PI3K/AKT pathway (196).

In female patients, menstrual disturbances are a common class of symptoms reported (197). However, these menstrual changes are thought to be transient and with no reported long-term consequences and could be attributed to immune response mediated stress (198). Cytokines and glucocorticoids have also been found in studies to effect on the hypothalamic-pituitary gonadal axis, arachidonic acid pathways, and the uterus, which may result in pregnancy-related unfavorable outcomes such as premature labor and miscarriages (199, 200). With these consequences mostly attributed to the inflammatory cytokine storm caused by SARS-CoV-2 infection, it is hypothesized that when inflammatory levels diminish post-COVID, these negative effects will as well.

One medicinal food which could be essential in the management of reproductive complications is *Rubus chingii*. *Rubus chingii* has traditionally been utilized in TCM to treat reproductive issues such as enuresis, impotence, and spermatorrhea (136). Although the exact mechanism of action has not been determined, animal studies have shown that using *Rubus chingii* in the TCM formula “Wu Zi Yan Zong Wan” increased sperm density, viability, and Ca^2+^ content in the sperm cytoplasm and mitochondria, thus improving overall sperm quality in males (137). Similarly, the use of *Rubus chingii* in a TCM formula called “He’s Yang Chao Recipe” was found to protect the ovaries from damage, reduce oxidative stress, and improve ovarian function in mice with primary ovarian insufficiency by inhibiting PINK1-Parkin mitophagy and NLRP3 inflammasome activation in female mice models (201).

### 4.7 Integumentary system

Amongst patients who have recovered from SARS-CoV-2 infection, several of them have reported dermatological issues. SARS-CoV-2 infection has been reported to cause telogen effluvium, which results in the most common dermatological complication reported being hair loss (38, 202). Lesions, ulcers, and blisters, for example, have been recorded as symptoms (203). Rash formation has also been reported from children recovering from SARS-CoV-2 infection. This could be mainly attributed to interactions between the SARS-CoV-2 spike protein and the basal epidermal cell ACE2 receptor (204, 205).

*Angelica dahurica* is a medicinal food that may be useful in the treatment of integumentary disorders. *Angelica dahurica* has long been used in TCM formulas to treat skin-related complications, and recent studies have shown that polysaccharides from *Angelica dahurica* have potent antioxidant effects as seen by their ability to inhibit lipid peroxidation, chelate Fe^2+^, and scavenge free radicals, which could be useful in managing inflammatory skin disorders such as rashes and lesions (83). Furthermore, Amygdalin, a major bioactive component of the herb *Armeniaca vulgaris*, was found to regulate the production of local pro-inflammatory cytokines primarily via the p38 MAPK/NF-κB signaling pathway, making it a good candidate to manage inflammatory skin diseases (85). *Prunus mume* combined with probiotics was also demonstrated to dramatically limit the formation of skin lesions while decreasing the peripheral eosinophil ratio and serum IgE concentrations (140).

Another key treatment goal is to inhibit the interactions between the SARS-CoV-2 spike protein and ACE2 receptors. According to a recent *in silico* study, the bioactive component Luteolin in *Portulaca oleracea* has a strong antiviral potential because it binds effectively to TMPRSS2, which, together with ACE2, is responsible for SARS-CoV-2 cell infection (138).

## 5 Insights and advances in medicinal food development

### 5.1 Technological advancements

The field of food science has expanded rapidly in recent years. One cause for this expansion might be the growing popularity of functional foods among health-conscious customers (206). As a result, a wide range of innovative food processing technologies have been explored and developed to alter or replace existing food processing procedures in order to provide higher-quality, more consumer-preferred foods (207). Some novel technologies include thermal technologies such as radio frequency heating, ohmic heating and microwave heating; and non-thermal technologies such as high pressure processing, ultrasound processing and cold pressure treatment (208).

**Figure 4:**
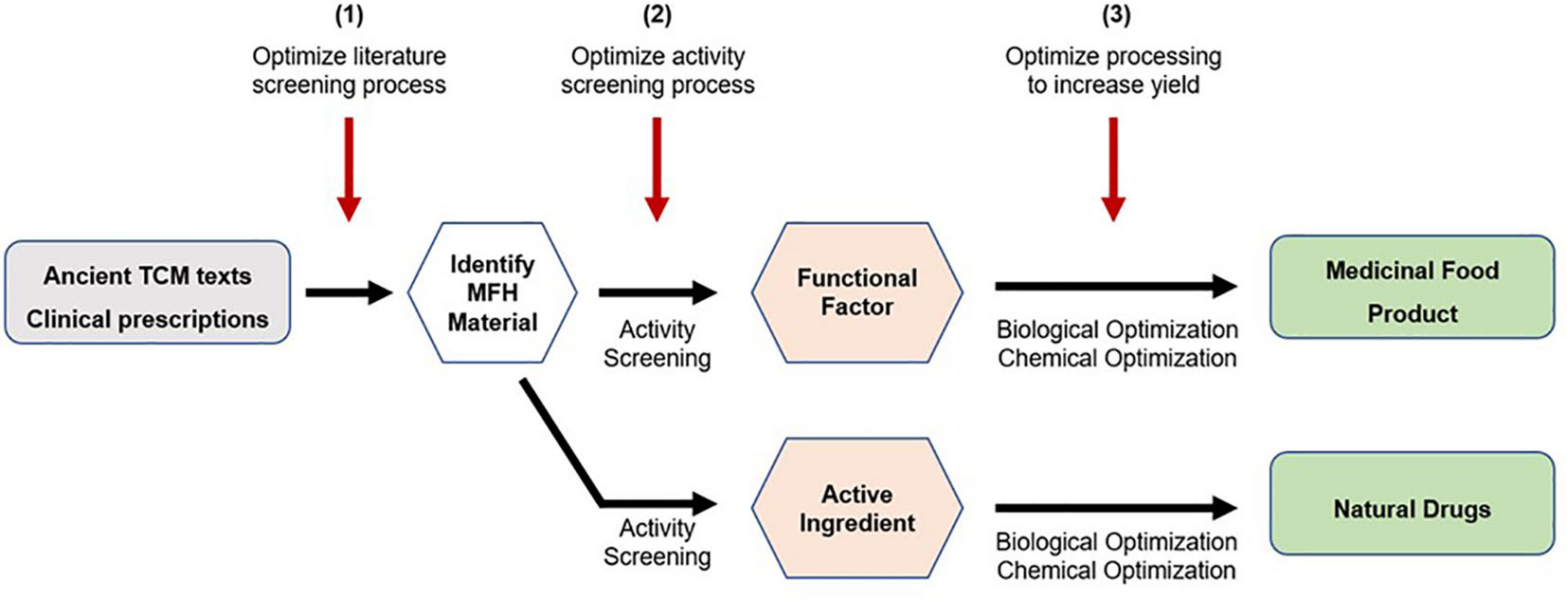
MFH development and potential areas of technological advancement.

The development of MFH medicinal foods typically involves the following steps (14). Firstly, raw herbs or medicinal plants are first identified through ancient texts and/or clinical prescriptions. Next, these raw materials will undergo activity screening to determine their functional factor and pharmacological bioactivities. Subsequently, these functional factors will undergo biological and chemical optimization processes to produce new medicinal food products. In some instances, the active compound can then be extracted, and optimized to produce drugs. An example will be the development of artemisinin as an anti-malarial drug from the Chinese medicinal plant *Artemisia annua* (209). In addition, the development of new and improved research techniques could be tapped on to optimize steps in the MFH development process. The first area of optimization would be the process of screening the literature for MFH materials. Text mining techniques such as information extraction, summarization, classification, clustering, and information visualization might be useful in successfully finding possible MFH materials (210). The activity screening procedure is the second area for improvement. In this regard, researchers might employ techniques such as cell culture, dialysis, ultrafiltration, chromatographic procedures, and target molecule immobilization to find active chemicals utilizing biological targets (211). Processing to boost yield would be the final area of optimization. In this regard, researchers should try to find the best conditions needed before utilizing improved drying processes to achieve maximal recovery of target bioactive chemicals for manufacture (212). A schematic diagram depicting the steps involved in MFH development, and potential areas of technological optimization is shown in **Figure 4** below.

Therefore, capitalizing on innovations in the food sector is critical for the future development of medical foods. Using new and enhanced technology to its maximum potential will allow for the optimization of the stages involved in the research and development of MFH medicinal foods. With enhanced processing technologies, this also creates opportunities to venture into larger-scale commercial manufacturing.

### 5.2 Development of new medicinal foods

The recent years have seen the development of several new medicinal food products (213). For instance, *Cordyceps sinensis*, an endoparasitic fungus, has gained favor as a tonic food due to claims of anti-cancer and anti-aging qualities (214). *Ganoderma lucidum*, more commonly known as “Lingzhi”, is another renowned herb that has been used for the prevention and treatment of bronchitis, allergies, hepatitis, immunological diseases, and cancer (215). Medicinal foods have also been derived from animals in our environment. For example, sea cucumber, a common TCM component, includes a high concentration of triterpene glycosides, which have antifungal, antiviral, and hemolytic properties (216). Therefore, the usage of TCM medicinal foods is growing more common, and the variety of medicinal foods for consumption is expanding as well.

According to previous studies, our environment contains over 3600 edible insect species and up to 300,000 different types of wild plants, the majority of which are edible (217). Only a handful of them, however, have been processed into other food items. Similarly, there are around 2000 different varieties of edible fungus in nature, but only 40 to 50 of them have been produced on a large scale artificially (14, 218). Hence, new edible elements with therapeutic characteristics are certain to be discovered, which may also be used in large-scale manufacture of medical food items. To broaden the scope of medicinal food manufacturing, research might focus on the discovery and screening of potential MFH materials.

## 6 Conclusions

Even though COVID-19 is no longer a public health emergency, people are still reporting post-COVID problems. There is a progressive shift from COVID disorders to post-COVID disorders, and there is a growing need to find safe and effective therapeutic approaches to address the negative consequences of post-COVID disorders. TCM is a valuable source of therapy options. There are certain plants designated as MFH among the herbs in the TCM pharmacopoeia. These plants are more commonly known as medicinal foods, and they can be ingested as a medication or as a food item. Due to their flexibility and safety in use, they are a suitable therapy option to explore in the long-term management of post-COVID disorders.

Because post-COVID disorders tend to remain longer and may develop to chronic conditions, MFH medicinal foods are a valuable resource in therapy, especially for chronic diseases that need a longer duration of medication use. Among the identified post-COVID problems, they mostly impact the cardiovascular, pulmonary, gastrointestinal, neurological, nephrological, reproductive, and integumentary systems of the body. One important aspect of therapy is reducing inflammation, and some TCM medicinal herbs have been demonstrated to have anti-inflammatory characteristics, making them ideal for treatment. As an MFH medical food, it is also safe to consume and may be included into one’s everyday diet for long-term usage.

With a greater emphasis on food science research, this might also benefit the large-scale development of MFH. The development of new techniques and technologies has accelerated in recent years, and the development of MFH therapeutic foods may benefit from this trend as well. Data science and related *in silico* approaches have also been enhanced and may be used to accelerate the discovery of potential MFH materials. Optimization might also be done on processes such as literature screening, activity screening, and processing to increase product yield.

For this study, we have chosen to focus on TCM medicinal herbs from the list of MFH items were reviewed. A total of 34 possible MFH herbs have been identified for the management of different post-COVID disorders based on the body systems affected. Other complementary and alternative medicine, such as Ayurvedic medicine, may give other types of MFH in the treatment of post-COVID problems in addition to TCM. Therefore, future research could focus on discovering other therapeutic plants utilized in complementary and alternative medicine.

In closing, our present review discussed the usage of TCM medicinal foods and their mechanisms of action in the management of post-COVID disorders and its complications. As the post-COVID situation is relatively new, future research could focus on elucidating the mechanisms of action of MFH medicinal foods in the treatment of post-COVID disorders, to translate positive findings from *in vitro* and *in vivo* models to human models. Furthermore, even though this present review focuses on the management of post-COVID disorders, the principles and potential herbs discussed here might be extended to the management of other respiratory problems with comparable pathogenic factors. Collectively, we hope that further research will pave the way for the large-scale creation of medical foods made from MFH materials for the benefit of society’s health.

## Author contributions

CN: Conceptualization, Writing - Original Draft, Writing - Review & Editing. HB: Supervision, Writing - Review & Editing. YZ: Conceptualization, Supervision, Writing - Review & Editing. LZ: Conceptualization, Supervision, Writing - Review & Editing. All authors contributed to the article and approved the submitted version.
